# *Cinnamomum verum* extract as a potential therapeutic for atopic dermatitis through reducing lipid peroxidation and oxidative stress

**DOI:** 10.3389/fphar.2025.1714816

**Published:** 2026-01-12

**Authors:** Min Jung Kim, Mi Jin Jang, Ju-Hye Yang, Young Zoo You, Ye Jin Yang, Ji Woong Heo, Han Nim Choi, Ryounghoon Jeon, Sang-Hyun An, Kwang Il Park

**Affiliations:** 1 College of Veterinary Medicine, Gyeongsang National University, Jinju, Republic of Korea; 2 Preclinical Research Center, Daegu Gyeongbuk Medical Innovation Foundation (K-MEDI hub), Daegu, Republic of Korea; 3 Korean Medicine (KM) Application Center, Korea Institute of Oriental Medicine, Daegu, Republic of Korea

**Keywords:** 4-hydroxynonenal, atopic dermatitis, Cinnamomum verum J. presl. [lauraceae], LC-QTOF-MS-MS, lipid peroxidation, oxidative stress

## Abstract

**Background:**

Oxidative stress and lipid peroxidation play crucial roles in the pathogenesis of atopic dermatitis (AD). Among the by-products of lipid peroxidation, 4-hydroxynonenal (4-HNE) is known to exacerbate inflammatory responses.

**Methods:**

This study investigated the potential of *Cinnamomum verum* J. Presl. [Lauraceae] extract (CVE) in mitigating oxidative stress and suppressing 4-HNE–mediated inflammatory pathways in an AD model. The antioxidant properties of CVE were evaluated through lipid peroxidation assays, reactive oxygen species (ROS) measurement, and cytokine profiling.

**Results:**

CVE treatment significantly reduced 4-HNE levels, decreased pro-inflammatory cytokine expression, and restored antioxidant enzyme activity.

**Conclusion:**

These findings suggest that CVE may serve as a natural therapeutic agent for managing oxidative stress and inflammation in AD.

## 1 Background

Atopic dermatitis (AD) is a long-lasting skin disorder marked by heightened immune reactions and oxidative stress (Xie et al., 2024). Lipid peroxidation, leading to the production of reactive aldehydes such as 4-Hydroxynonenal (4-HNE), is considered a significant mechanism involved in the pathogenesis of AD. The aldehydes initiate the activation of the nuclear factor kappa B (NF-κB) signaling pathway, resulting in the induction of pro-inflammatory mediators, such as cyclooxygenase-2 (COX-2) and inducible nitric oxide synthase (iNOS) (Yamamoto and Gaynor, 2001). The increase in 4-HNE levels exacerbates the inflammatory response and tissue impairment, establishing a self-perpetuating cycle of oxidative stress and inflammation (Ansari et al., 2021). Reactive oxygen species (ROS) produced during the inflammatory process initiate lipid peroxidation, causing the secretion of pro-inflammatory mediators such as prostaglandins and arachidonic acid (Su et al., 2019). These lipid oxidation products have the potential to function as damage-associated molecular patterns, which subsequently trigger the NF-κB pathway and enhance inflammatory reactions (Uchida, 2013). Specifically, 4-HNE is recognized for its ability to interact with cellular proteins, resulting in structural impairment and the generation of further inflammatory mediators (Shoeb et al., 2014). Aldehyde dehydrogenase 2 (ALDH2) is essential for the metabolism of reactive aldehydes, including 4-HNE, during aldehyde detoxification (Jaganjac et al., 2020). Nevertheless, when exposed to oxidative stress, ALDH2 function is frequently hindered, resulting in the buildup of 4-HNE and the subsequent initiation of inflammatory pathways (Li et al., 2022).

Despite the central role of corticosteroids in AD management, their long-term use is limited by adverse effects such as skin atrophy, impaired wound healing, and suppression of immune responses (DiRuggiero and DiRuggiero, 2025). These drawbacks highlight the unmet need for alternative strategies that can effectively control inflammation and oxidative stress with a lower risk of toxicity (Siegfried et al., 2016). In this context, natural product-based interventions such as *Cinnamomum verum* J. Presl. [Lauraceae] extract (CVE) have gained increasing attention as potential adjuncts or substitutes for conventional therapies.

*Cinnamomum verum* J. Presl. [Lauraceae] (CV), also referred to as true cinnamon, contains high levels of polyphenols and flavonoids that exhibit potent antioxidant and anti-inflammatory properties (Pagliari et al., 2023). Previous studies have demonstrated that *C. verum* exerts beneficial effects in models of metabolic syndrome, cardiovascular disorders, and neuroinflammation, largely through the modulation of oxidative stress pathways (Mollazadeh and Hosseinzadeh, 2016; Mohammadabadi and Jain, 2024). However, evidence regarding its efficacy in skin inflammatory diseases such as AD remains scarce. In particular, whether CVE can attenuate 4-HNE accumulation and restore ALDH2 function, a key mechanism of lipid peroxidation detoxification, has not been systematically investigated. Addressing this knowledge gap is crucial, as 4-HNE–mediated ALDH2 dysfunction represents a pivotal driver of chronic oxidative damage and inflammation in AD.

Hence, increasing ALDH2 activity or suppressing the synthesis of 4-HNE is viewed as a hopeful approach for the treatment of inflammation-associated diseases like AD. Nevertheless, current treatments for AD, such as corticosteroids, while effective in reducing inflammation, are associated with adverse outcomes such as epidermal thinning, telangiectasia, and increased susceptibility to secondary infections (Barta et al., 2023). By contrast, natural products such as CVE, enriched with polyphenols and flavonoids, are generally recognized for their favorable safety profile and lower systemic toxicity, making them more suitable for chronic management (Thakurdesai et al., 2024). Although direct comparisons are limited, these pharmacological features suggest that CVE may provide therapeutic benefits with fewer safety concerns than corticosteroid-based regimens.

Nevertheless, the precise mechanism in which CV extract (CVE) diminishes lipid peroxidation and regulates inflammation induced by 4-HNE is still uncertain. Thus, the objective of this study is to assess the efficacy of CVE for decreasing levels of reactive oxygen species, inhibiting 4-HNE production, and enhancing ALDH2 activity in order to prevent NF-κB activation and the expression of pro-inflammatory mediators such as COX-2 and iNOS. In contrast, natural products like CVE may offer safer AD treatment with fewer side effects and lower toxicity, making them suitable for long-term management of chronic inflammatory diseases. Furthermore, Liquid Chromatography Quadrupole Time-of-Flight Tandem Mass Spectrometry (LC-QTOF-MS-MS) analysis will be utilized to detect plant metabolites found in CVE and verify their potential ability to inhibit oxidative stress and lipid peroxidation *in vitro*. This study aims to evaluate the potential of CVE in mitigating ROS-induced lipid peroxidation and suppressing NF-κB-mediated inflammation in HaCaT cells and an animal model of AD. We will also assess whether CVE enhances ALDH2 activity to reduce the accumulation of 4-HNE and downregulates pro-inflammatory mediators such as COX-2 and iNOS.

## 2 Materials and methods

### 2.1 Extraction and sampling

CV cortex (50 g) was steeped in 1,000 mL of spring water (White, Muhaksan Cheongsaemmul Co., Ltd., Sancheong-gun, Gyeongsangnam-do, Korea) for 1 h in a decoction extractor (Daewoong DWP-5000M), followed by hot water extraction via heating for 3 h. The resulting extract was filtered through a 106 μm test sieve (No. 140, Retsch, Germany). The filtered extract was then frozen in a deep freezer and subsequently lyophilized to prepare the sample. Finally, the abstracts obtained were named ‘CVE’ and were kept at −20 °C until further use (Yield: 4.64%).

### 2.2 Total polyphenol and total flavonoid content

The total polyphenol and flavonoid contents of CVE were determined using Folin-Ciocalteu and aluminum chloride colorimetric methods, respectively. For polyphenol content measurement, gallic acid (Sigma-Aldrich, St. Louis, MO, United States) solutions (1 mg/mL in distilled water) were prepared and diluted to generate a calibration curve. A mixture containing 100 μL of CVE (2 mg/mL) or gallic acid standard, 500 μL of 2N Folin’s phenol reagent (Sigma-Aldrich, St. Louis, MO, United States), and 400 μL of 7.5% sodium carbonate (Na_2_CO_3_) was prepared. After mixing thoroughly, the samples were incubated in the dark at room temperature for 1 h. Absorbance was measured at 750 nm using a SYNERGY H1 microplate reader (BioTek, Winooski, Vermont, United States). Results were expressed as mg gallic acid equivalents (GAE) mg/g based on the calibration curve. For flavonoid content measurement, quercetin (Sigma-Aldrich, St. Louis, MO, United States) solutions (1 mg/mL in 100% methanol) were prepared and used to generate a calibration curve. A mixture containing 100 μL of CVE (2 mg/mL) or quercetin standard, 860 μL of 80% ethanol, 20 μL of 10% aluminum chloride (AlCl_3_), and 20 μL of 1 M potassium acetate (BIOSTEM, Seoul, Republic of Korea) was prepared. The samples were incubated at room temperature for 40 min, and absorbance was measured at 415 nm using the SYNERGY H1 microplate reader. Results were expressed as mg quercetin equivalents (QE) mg/g.

### 2.3 Measurement of antioxidant efficacy

To evaluate the free radical scavenging ability of CVE, the DPPH and ABTS assays were performed with modifications. DPPH (Thermo Fisher Scientific, Waltham, MA, United States) is employed as a reagent to provide a simple and precise method for titrating the oxidizable groups of natural or synthetic antioxidants. Ascorbic acid (AC, Sigma-Aldrich, St. Louis, MO, United States) was used as a standard antioxidant (positive control). A volume of 190 μL of 0.2 mM DPPH methanolic solution was added to each well of a 96-well plate, followed by the addition of 10 μL of the sample, 100 μg/mL AC, or solvent for blank controls. The mixture was incubated at 37 °C for 30 min, and absorbance was measured at 517 nm using a microplate reader (BioTek, Winooski, VT, United States). The concentration of the blue-green ABTS (Sigma-Aldrich, St. Louis, MO, United States) radical solution was adjusted with methanol to achieve an absorbance of 0.70 ± 0.04 at 734 nm. ABTS radicals were generated through an oxidation reaction involving potassium persulfate (Sigma-Aldrich, St. Louis, MO, United States). To ensure freshness, radical stock solutions were prepared immediately before use. The reaction was carried out in the dark at 20 °C for 5 min.

### 2.4 Cell culture maintenance

HaCaT cells, a human keratinocyte cell line, were acquired from the Korea Institute of Oriental Medicine and sourced from Cell Lines Service (Eppelheim, Deutschland, Germany). The cell culture media, Dulbecco’s Modified Eagle’s Medium supplemented with 10% heat-inactivated fetal bovine serum (Thermo Fisher Scientific, MA, United States) and 1% penicillin/streptomycin was purchased from Gibco (Thermo Fisher Scientific, MA, United States). In this study, cells were used only after undergoing a minimum of three passages in order to promote cellular stability and reduce the potential variability linked to lower passage numbers.

### 2.5 HaCaT cell viability

Cell viability was assessed through the implementation of the MTT (#298-93-1, Duchefa Biochemie, Haarlem, Netherlands) assay. The cells (1 × 10^4^ cells/well) were seeded in 96 well plates and stabilized for 24 h. Cells were treated with CVE at various concentrations, and cell viability was subsequently evaluated 3 times. The HaCaT cells were incubated with concentration from 10 to 300 μg/mL of CVE for 24 h. The wells were gently washed, and the medium replaced with MTT reagent for 2 h. The absorbance was then measured with a BioTex Fluorescence/multimode microplate reader (Agilent Technologies, Santa Clara, CA, United States) at 565 nm.

### 2.6 Evaluation of intracellular ROS production by DCF-DA assay in HaCaT cell

HaCaT cells were cultured a 6-well plate 5 × 10^5^ cells/well and allowed to stabilize for 24 h 37 °C, in 5% CO_2_ conditions. Cells were stained with ROS-ID Total ROS (Enzo Life Sciences, Farmingdale, NY, United States). DCF-DADCF-DA (Thermo Fisher Scientific, Waltham, MA, United States) was added and incubated for 1 h before fluorescence was measured using a BioTek Synergy multimode microplate reader (Agilent Technologies, Santa Clara, CA, United States) and a BioTek Cytation 7 imaging multimode reader (Agilent Technologies, Santa Clara, CA, United States) (Kim et al., 2025).

### 2.7 TBARS (thiobarbituric acid reactive substances) assay

The impact on lipid peroxidation within cells was evaluated through the TBARS assay. HaCaT cells were subjected to a specific amount of UVB (50 mJ/cm^2^) radiation in order to elicit lipid peroxidation. UVB irradiation was performed using the BLX 312 UV irradiation system (Vilber Lourmat, Germany). The irradiation distance was approximately 15 cm, which is the distance between the UVB light source, and the plate placed on the floor. Cells were irradiated with an intensity of 50 mJ/cm^2^ and a UVB wavelength range of 312 nm.

The supernatant was then collected and processed using an oxidative stress assay kit according to the method provided by the manufacturer, and the absorbance was measured at 532 nm to measure and quantify free malondialdehyde (MDA), a representative indicator of lipid peroxidation.

### 2.8 Protein extraction and western blotting

We then evaluated protein levels produced by cells exposed to extracts (Yang et al., 2024a). To do so, HaCaT cells were first seeded at 1 × 10^6^ cells/well in a 60π dish. After 24 h, CVE and Tumor Necrosis Factor-alpha (TNF-α, NKMAX, Seongnam, Republic of Korea)/Interferon-gamma. (IFN-γ, NKMAX, Seongnam, Republic of Korea) were both added and the resulting mixture was incubated for 24 h. Cells were washed with PBS (BioSeSang, Seoul, Republic of Korea) three times before being lysed with a protease inhibitor solution (100×, GenDEPOT, Katy, Texas, United States) suspended in a radio-immunoprecipitation assay buffer (Thermo Fisher Scientific, Waltham, Massachusetts, United States). Next, cell lysates were refrigerated centrifuge (1730R, GYROZEN Co., Ltd., Gimpo, Republic of Korea) at 13,000 rpm at 4 °C for 15 min. After centrifugation, the supernatants were transferred and protein levels were quantified using a bicinchoninic acid protein quantification kit (BioMax, Seoul, Republic of Korea). We then added a 5× sample buffer (ELPis, Daejeon, Republic of Korea) to the lysate containing 20 µg of protein and heated the resulting mixture at 80 °C for 15 min, after which equal amounts of protein from each sample were separated on an 8% SDS-PAGE gel. Proteins were then transferred to a polyvinylidene fluoride membrane (GVS filter technology, Sanford, Maine, United States) and blocked in 5% Bovine Serum Albumin in Tris-Buffered Saline (TBS) containing 0.1% Tween-20 (TBS-Tween; WB) for 2 h. After blocking, proteins were incubated with an appropriate primary antibody ALDH2 (#ATGA0450, NKMAX, Seoul, Republic of Korea), p-NF-κB (#3031, Cell Signaling Technology, MA Danvers, United States), p-IκBα (#2859, Cell Signaling Technology, MA Danvers, United States), COX-2 (#12282, Cell Signaling Technology, MA Danvers, United States), iNOS (#13120, Cell Signaling Technology, MA Danvers, United States), and β-actin (#ATGA0570, NKMAX, Seoul, Republic of Korea) overnight at 4 °C. After 24 h proteins were washed with WB and membranes were incubated with appropriate secondary antibodies for 2 h at 24 °C. Finally, protein bands were detected using a SuperSignal™ West Pico PLUS Chemiluminescent Substrate Kit (Thermo Fisher Scientific, Seoul, Republic of Korea) and analyzed using an imaging system (Shenhua Science Technology, Hangzhou, China).

### 2.9 LC-QTOF-MS/MS analysis conditions

The analysis of CVE was performed using a Nexera XS UHPLC system (Shimadzu, Kyoto, Japan), interfaced with an X500R QTOF-MS (SCIEX ExionLC AD system, Framingham, MA, United States) equipped with an electrospray ionization (ESI) source. For chromatographic separation, a Pronto SIL 120-5-C18 SH column (150 × 4.6 mm, 5 μm; Bischoff Chromatography, Leonberg, Germany) was utilized, with the column oven held at 35 °C (Yang et al., 2024b). The mobile phase was composed of solution A (0.1% formic acid in H_2_O) and solution B (0.1% formic acid in acetonitrile). The gradient elution was programmed as follows: the run began with 5% B and maintained this composition until 10 min. From 10 to 20 min, the proportion of solution B increased to 20%, followed by a gradual rise to 25% at 30 min. The concentration of B then increased to 40% by 40 min, and further to 70% at 55 min. A sharp increase to 95% B was implemented by 70 min. Finally, the gradient returned to 10% B at 75 min. The flow rate was maintained at 0.5 mL/min, and the injection volume was 2 μL. Plant metabolites identification via QTOF-MS was executed under positive ion mode with an ESI. All data processing and plant metabolites identification were conducted using SCIEX OS software (version 3.0.0.3339).

### 2.10 Animals and experiments

SKH-1 hairless mice (6 weeks old, 25 g–28 g) were purchased from Orient Bio (Seoul, Republic of Korea) and maintained under standard conditions (22 °C ± 3 °C and relative humidity 55% ± 5% under a 12 h light/dark cycle with food and water available *adlibitum*). All animal experiments procedures were reviewed beforehand and approved by the Institutional Animal Care and Use Committee of Daegu Gyeongbuk Medical Innovation Foundation (K-MEDI hub) (DGMIF-19043003-00). They were randomly divided into three groups: (1) Control group (normal skin, no AD induction), (2) AD-induced group (without treatment), and (3) CVE-treated AD group. Each group consisted of five mice, and five mice per group were evaluated at each time point (Days 3, 7, and 14). To induce AD, 2,4-dinitrochlorobenzene (DNCB), a known allergen, was used to trigger an immune response similar to human AD. On Day 0, the dorsal skin was shaved and cleaned. On Day 1, 200 μL of 1% DNCB in acetone/olive oil (3:1, v/v) was applied to the dorsal skin for initial sensitization. To maintain AD-like symptoms, 0.5% DNCB (200 μL) was applied every 3 days for 2–3 weeks and CVE 200 mg/kg was applied once a day for 6–7 weeks (Figure 3A). The severity of skin lesions was monitored and scored based on redness, swelling, dryness, excoriation (scratching), and crust formation.

### 2.11 Hematoxylin and eosin (H&E) staining

SKH-1 mouse were sacrificed after 3, 7, 14 days each. Dorsal skins of SKH-1 hairless mice were fixed in 10% formaldehyde (Sigma-Aldrich, St. Louis, MO, United States) for 24 h and embedded in paraffin wax. To measure changes in epidermal thickness, tissues were serially sectioned at 4 μm and stained with H&E. Epidermal thicknesses were determined using HK Basic software (KOPTIC, Seoul, Republic of Korea) in three randomly selected sections per mouse.

### 2.12 Immunohistochemistry (IHC) staining

For IHC, the sections were deparaffinized, rehydrated, and incubated in a 99 °C water. Then the sections were treated with H_2_O_2_ for peroxidase activity inhibition for 5 min and blocked with protein blocking serum for 1 h. Subsequently, the sections were incubated with primary antibody anti-4HNE (ab46545, 1:100) overnight at 4 °C. After rinsing with PBS, the sections were incubated with biotin-conjugated secondary antibody (1:200) for 1 h at room temperature. For detection, sections were incubated with ABC complex kit and then visualized using the VectorNovaRED substrate kit. The nuclei were counterstained with hematoxylin.

### 2.13 Statistical analysis

All *in vitro* experiments were conducted in triplicate (n = 3), and the data are expressed as the mean ± standard error of the mean (SEM). Statistical analyses were performed using GraphPad Prism version 8.0 (GraphPad Software, San Diego, California, United States). Differences among group means were evaluated using one-way analysis of variance (ANOVA), followed by Dunnett’s multiple comparison test for pairwise comparisons. A p-value <0.05 was considered statistically significant. For *in vivo* experiments, each experimental group consisted of animals (N = 5). Data are presented as mean ± SEM, and statistical significance was determined using one-way ANOVA with Dunnett’s multiple comparison test. A p-value <0.05 was considered statistically significant.

## 3 Results

### 3.1 Antioxidant properties of CVE and its protective effects against oxidative stress in HaCaT cells

**FIGURE 1:**
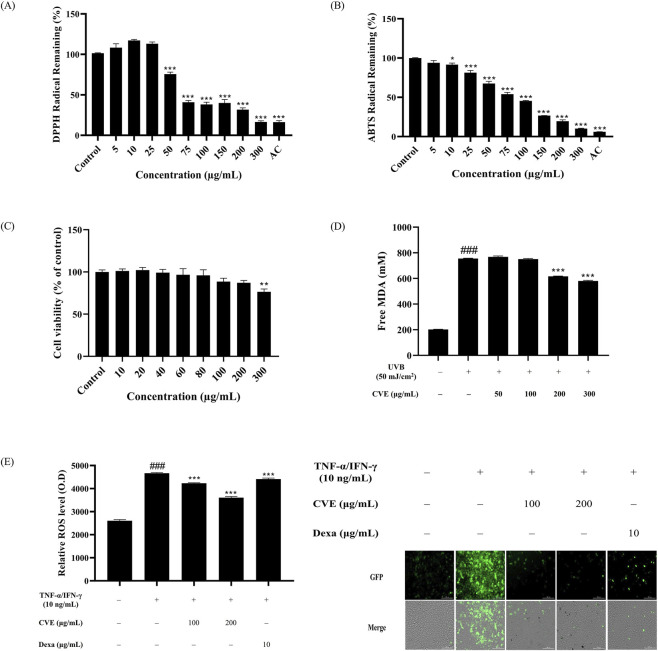
CVE exhibits antioxidant effects and protects HaCaT cells against oxidative stress. **(A)** DPPH radical remaining and **(B)** ABTS radical remaining, using ascorbic acid (AC) as a positive control. **(C)** Effects of various concentrations of CVE on HaCaT cell viability for 24 h ^*^p < 0.05; ^**^p < 0.01; ^***^p < 0.001 vs. untreated control. **(D)** Measurement of malondialdehyde (MDA) levels to assess lipid peroxidation. **(E)** Quantitative analysis of fluorescence intensity demonstrates that CVE treatment significantly reduces oxidative stress and fluorescence staining images show oxidative damage. Data represents the mean ± SEM. ^###^p < 0.001 vs. untreated control; ^***^p < 0.001 vs. TNF-α/IFN-γ -treated group. Scale bar: 200 µm.

We first measured CVE total polyphenol and flavonoid to assess the antioxidant effect. The total polyphenol was found to be 57.917 ± 0.46 GAE mg/g, while the total flavonoid content was 46.296 ± 0.849 QE mg/g in CVE (Table 1). An DPPH and ABTS assay were performed to measure the antioxidant effect of CVE using 100 μg/mL AC as a positive control. CVE was tested at concentrations of 5, 10, 25, 50, 75, 100, 150, 200, and 300 μg/mL. Our results showed that CVE exerted a strong antioxidant effect in a dose-dependent manner (Figures 1A,B).

**TABLE 1:** Total phenolic and flavonoid contents of CVE.

Items		Concentration
CVE	Total polyphenol (GAE mg/g)^1^	57.917 ± 0.46
	Total flavonoid (QE mg/g)^2^	46.296 ± 0.849

1Total polyphenol content is expressed as gallic acid equivalents (GAE).

2Total flavonoid content is expressed as quercetin equivalent (QE).

We then measured the cytotoxicity of CVE against HaCaT cells using a MTT assay. We found that CVE showed no cytotoxicity over a range of concentrations (i.e., 10, 20, 40, 60, 80, 100 and 200 μg/mL). Our results showed that 300 μg/mL showed cytotoxic effects. These results confirmed that CVE is a safe substance for use in <300 μg/mL this study (Figure 1C). HaCaT cells were pre-treated with different concentrations of CVE before being subjected to UVB irradiation to induce lipid peroxidation and oxidative stress. The degree of lipid peroxidation induced by UVB was evaluated through the TBARS assay to assess the effectiveness of CVE in inhibiting lipid oxidation. The findings indicate that CVE effectively inhibited lipid peroxidation, suggesting a potential protective role against UVB-induced oxidative damage. The level of lipid peroxidation is indicated by the fluorescence intensity, with higher levels of intensity indicating greater oxidative damage. The group that received the treatment exhibited a notable decrease in lipid peroxidation, indicating that the use of CVE may have the ability to suppress oxidative stress. (Figure 1D). The fluorescence images indicate that treatment with TNF-α/IFN-γ induces significant oxidative stress, as shown by the increased green fluorescence signal. However, the groups pretreated with CVE exhibited a reduction in fluorescence intensity, suggesting that CVE effectively alleviates TNF-α/IFN induced oxidative stress in HaCaT cells (Figure 1E).

### 3.2 CVE activates ALDH2 and inhibits NF-κB signaling in TNF-α/IFN-γ induced oxidative stress conditions

**FIGURE 2:**
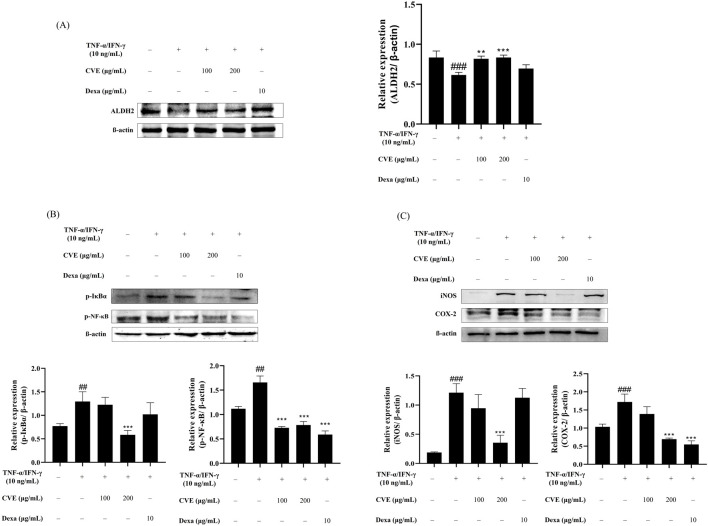
CVE inhibits NF-κB activation and pro-inflammatory protein expression through ALDH2 activation. **(A)** ALDH2 expression levels were analyzed by Western blotting in HaCaT cells treated with TNF-α/IFN-γ and CVE. **(B)** NF-κB pathway activation was assessed by analyzing the phosphorylation levels of IκBα and p65 by Western blotting in HaCaT cells treated with TNF-α/IFN-γ and CVE. **(C)** COX-2 and iNOS expression levels were evaluated by Western blotting in HaCaT cells treated with TNF-α/IFN-γ and CVE. Data represent the mean ± SEM. ^###^p < 0.001 vs. untreated control. ^**^p < 0.01; ^***^p < 0.001 vs. TNF-α/IFN-γ -treated group.

CVE effectively upregulated ALDH2 expression in a dose-dependent manner (Figure 2A). In the TNF-α/IFN-γ-treated group, NF-κB activation was significantly increased, as evidenced by the phosphorylation of IκBα and NF-κB (Figure 2B). However, CVE treatment suppressed this phosphorylation, suggesting that CVE inhibits NF-κB activation. Furthermore, the expression of pro-inflammatory proteins COX-2 and iNOS was significantly reduced in the CVE-treated group (Figure 2C). These results indicate that CVE alleviates TNF-α/IFN-γ induced inflammation by activating ALDH2 and subsequently inhibiting NF-κB signaling.

### 3.3 Effect of CVE on skin inflammation and Clinical Skin Index in AD-induced SKH-1 mice

**FIGURE 3:**
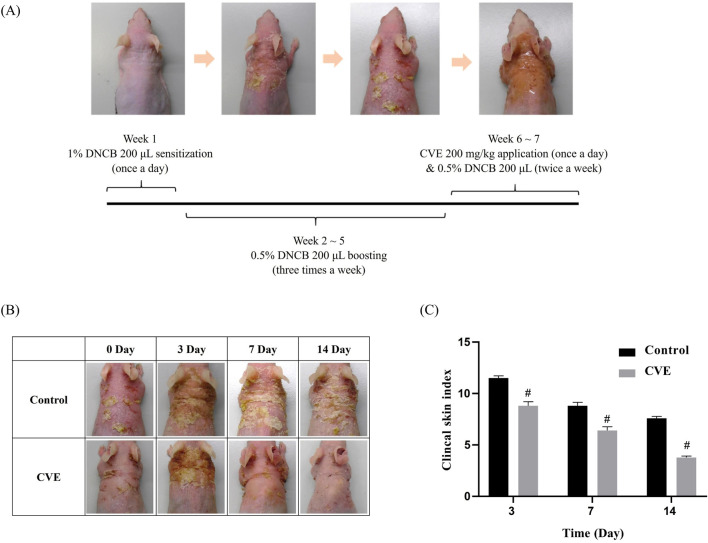
Effects of CVE on DNCB-Induced AD in SKH-1 mouse **(A)** Scheme of experiment designs **(B)** effect of CVE on clinical skin features of DNCB-induced AD in SKH-1 mouse **(C)** Clinical skin index of AD in SKH-1 mouse (N = 5) (^#^p < 0.05).

Skin lesions characterized by scaling, redness, and inflammation are present. Over time, the CVE-treated group shows a noticeable reduction in inflammation and improvement in skin condition compared to the control group. The Clinical Skin Index (Right Panel) reveals that the control group (represented by the black bar) maintains a higher skin index throughout the observation period. In contrast, the CVE-treated group (depicted by the gray bar) demonstrates a significant decrease in the Clinical Skin Index over time. The difference between the two groups becomes more pronounced on Day 7 and Day 14, with statistical significance (p < 0.05, indicated by asterisks), suggesting that CVE effectively alleviates skin inflammation (Figures 3B, C).

### 3.4 Effect of CVE on epidermal thickness and inflammatory response in AD-induced SKH-1 mice

**FIGURE 4:**
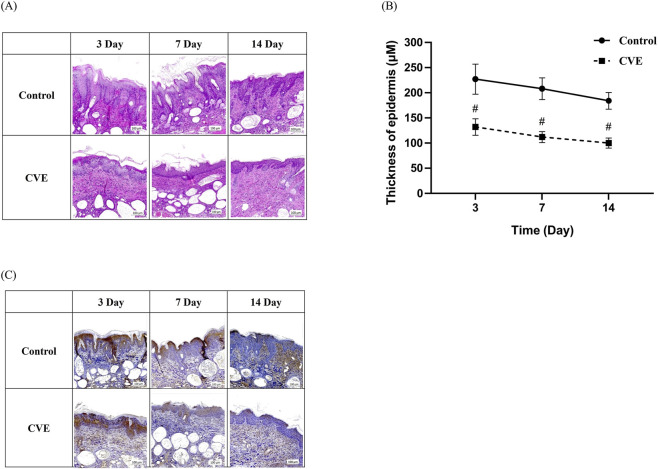
Effect of CVE on epidermis thickness change in dorsal skin tissue of DNCB-induced AD in SKH-1 mouse. **(A)** H&E staining and **(B)** thickness of epidermis **(C)** Effect of CVE on 4HNE change by IHC staining in dorsal skin tissue of DNCB-induced AD in SKH-1 mouse. (^#^p < 0.05) Scale bar:100 µm.

To evaluate the anti-inflammatory and skin barrier-improving effects of CVE, H&E staining and IHC analysis were performed on AD-induced mouse skin tissue. Compared to the control group, the CVE-treated group showed a significant reduction in epidermal thickness, indicating improved skin condition. The epidermal thickness was measured on days 3, 7, and 14, and the CVE-treated group exhibited a gradual decrease in epidermal thickness over time. This suggests that CVE alleviates skin inflammation and prevents abnormal epidermal hyperplasia (Figure 4A). The epidermal thickness measurement graph demonstrates that the CVE-treated group exhibited a significant reduction in epidermal thickness compared to the control group (*p* < 0.05), especially from day 7 onwards (Figure 4B). IHC staining was conducted to assess the expression of pro-inflammatory cytokines and oxidative stress markers. The CVE-treated group showed reduced expression of inflammatory markers, indicating that CVE suppresses inflammation and oxidative damage in the epidermis (Figure 4C). This confirms that CVE effectively reduces inflammation and improves skin barrier function in an AD-induced mouse model.

### 3.5 LC-QTOF-MS-MS analysis of CVE components

**FIGURE 5:**
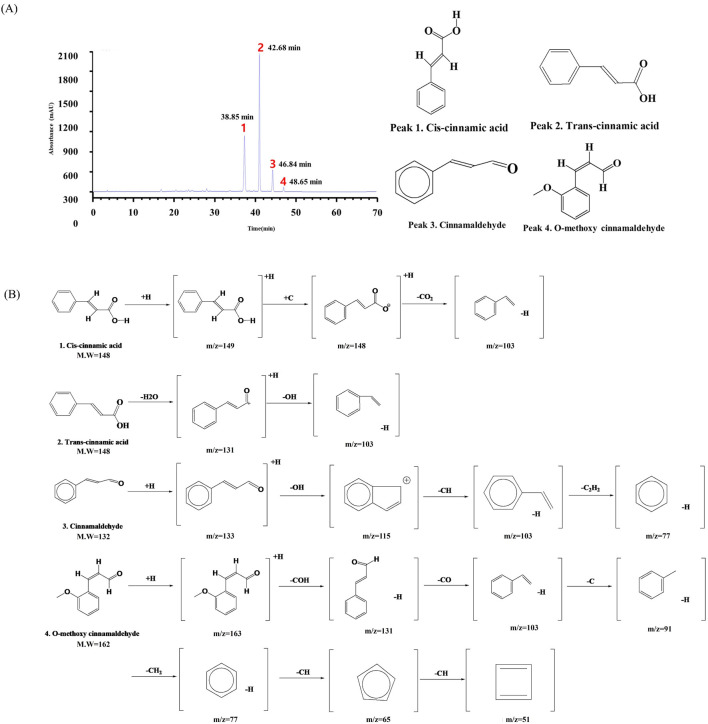
Liquid Chromatography Quadrupole Time-of-Flight Mass Spectrometry (LC-QTOF-MS-MS) analysis of CVE. **(A)** The chromatogram illustrates the retention times of key compounds at 284 nm, **(B)** while the mass spectra depict their corresponding fragmentation patterns.

The LC-QTOF-MS-MS analysis identified key plant metabolites present in CVE (Supplementary Figure S1). Four distinct plant metabolites Cinnamic acid (Redeuil et al., 2009), Trans-cinnamic acid (Redeuil et al., 2009), Cinnamaldehyde (Tambe and Gotmare, 2022), O-methoxy cinnamaldehyde (Thapa et al., 2021), were successfully identified (Peaks 1–4, Figure 5A). The retention times of these plant metabolites were as follows: 38.85 min (Cis-cinnamic acid), 42.68 min (Trans-cinnamic acid), 46.84 min (Cinnamaldehyde), 48.65 min (O-methoxy cinnamaldehyde) (Supplementary Figures S2-S4; Table 2). Their molecular structures are depicted in Figure 5B.

**TABLE 2:** Tentative identification of the chemical components of the CVE obtained from the UPLC-TOF-MS/MS analysis.

Peak no.	Plant metabolites	Molecular formula (g/mol)	Retention time (min)	MS Fragment (m/z) [M + H]
1	Cis-cinnamic acid	C_9_H_8_O_2_ (148.16 g/mol)	38.85	149, 148, 103
2	*Trans*-cinnamic acid	C_9_H_8_O_2_ (148.16 g/mol)	42.68	149, 131, 103
3	Cinnamaldehyde	C_9_H_8_O (132.16 g/mol)	46.84	133,115,103, 77
4	O-methoxy cinnamaldehyde	C_10_H_10_O_2_ (162.18 g/mol)	48.65	163, 131, 103, 91, 77, 65, 51

## 4 Discussion

ROS and lipid peroxidation are acknowledged as factors in the onset of inflammatory skin conditions such as AD (Briganti and Picardo, 2003). Studies have shown a growing focus on the link between oxidative stress and AD, with findings suggesting elevated levels of MDA, an important biomarker of lipid peroxidation (Sivaranjani et al., 2013; Tsukahara et al., 2003). Oxidative stress occurs when there is an imbalance between the excessive production of reactive oxygen species and inadequate levels of antioxidants, disrupting the equilibrium maintained by antioxidant plant metabolites (Jomova et al., 2023). Various natural antioxidants, such as *Rubus coreanus*, *Acer tegmentosum* Maxim., and *Rosmarinus officinalis*, are listed with the Ministry of Food and Drug Safety (Korea, 2017). These naturally occurring plant metabolites, which commonly contain high levels of polyphenols and flavonoids, are being increasingly studied as potential substitutes for synthetic antioxidants in combating oxidative stress and inflammation (Hussain et al., 2016).

Oxidative stress contributes to AD pathogenesis by disrupting keratinocyte function and the skin barrier, and by promoting inflammation (Raimondo et al., 2023). Lipid peroxidation products such as MDA and 4-HNE accumulate under these conditions. MDA serves as a general marker, while the more reactive 4-HNE forms adducts with proteins and directly exacerbates inflammation (Barrera et al., 2018).

The total polyphenol and flavonoid content of CVE were quantified, revealing substantial levels of these plant metabolites. The high polyphenol and flavonoid content corresponded with the strong antioxidant capacity of CVE, as evidenced by DPPH and ABTS assays (Table 1; Figures 1A,B).

MDA is a compounds generated through the peroxidation of polyunsaturated fatty acids (Zhu et al., 2024). It has been widely utilized as a biomarker for assessing oxidative stress in various biological samples from patients afflicted with a broad spectrum of diseases (Cordiano et al., 2023). In UVB-irradiated HaCaT cells, CVE exhibited a protective effect against oxidative stress-induced lipid peroxidation. The TBARS assay confirmed that CVE treatment significantly reduced lipid peroxidation levels, highlighting its potential to counteract oxidative damage (Figure 1D). Additionally, fluorescence imaging demonstrated that CVE reduced TNF-α/IFN-γ-induced oxidative stress in HaCaT cells (Figure 1E). These findings support the notion that CVE exerts cytoprotective effects by neutralizing ROS and preserving cellular integrity.

External stimuli such as UVB or TNF-α/IFN-γ can induce oxidative stress, which in turn can facilitate the production of ROS and subsequently result in lipid peroxidation (Punnonen et al., 1991; Frances et al., 2013). This process leads to damage of cellular membranes and the buildup of toxic aldehydes, such as MDA and 4-HNE (Barrera et al., 2018). These aldehydes interact with proteins and DNA, leading to cellular dysfunction and exacerbating inflammatory reactions (Voulgaridou et al., 2011). Aldehyde dehydrogenase (ALDH) plays a vital role in the detoxification process by facilitating the elimination of harmful aldehydes (Jin et al., 2022). ALDH2 acts protectively by breaking down endogenous aldehydes, such as acetaldehyde, in the mitochondria (Li et al., 2024). Elevated ALDH2 activity has the potential to efficiently remove lipid peroxidation products that are triggered by ROS, subsequently sustaining the cellular redox equilibrium and reducing cellular harm (Gao et al., 2022).

Previous studies mainly focused on antioxidant effects of natural products in reducing ROS or MDA (Muchtaridi et al., 2024). Here, we show that CVE enhances ALDH2-mediated detoxification of 4-HNE, linking lipid peroxidation directly to inflammatory regulation in AD, and providing a more precise target for therapy.

Western blot analysis revealed that CVE upregulated ALDH2 expression in a dose-dependent manner, suggesting that CVE enhances aldehyde detoxification pathways (Figure 2A). Furthermore, CVE suppressed NF-κB activation by inhibiting the phosphorylation of IκBα and p65, leading to a significant reduction in the expression of pro-inflammatory mediators COX-2 and iNOS (Figures 2B,C). ALDH2 has been documented to inhibit NF-κB activation through the regulation of TNF-α signaling (Legler et al., 2003). This implies that higher levels of ALDH2 expression could potentially help reduce inflammation. Hence, the documented inhibition of ROS generation and upregulation of ALDH2 expression by CVE may serve as a significant process in mitigating inflammation through the suppression of the NF-κB signaling pathway, as well as facilitating the elimination of harmful aldehydes.

Elevated 4-HNE and MDA in skin associate with barrier dysfunction, keratinocyte damage, and pruritus, hallmark symptoms of AD (Wroński et al., 2023). MDA is a general lipid peroxidation marker, whereas reactive 4-HNE exacerbates inflammation (Barrera et al., 2018). By reducing these aldehydes, CVE not only confirms their biomarker role but also demonstrates a mechanism for targeted intervention.

The *in vivo* findings in DNCB-induced AD mice further supported the anti-inflammatory effects of CVE (Figure 3A). Mice treated with CVE exhibited noticeable improvements in clinical skin features, including reductions in scaling, redness, and inflammation. The Clinical Skin Index was significantly lower in the CVE-treated group, indicating that CVE effectively alleviates AD symptoms (Figure 3B). Additionally, histological analysis revealed that CVE treatment reduced epidermal thickness and suppressed inflammatory marker expression in the skin, suggesting that CVE enhances skin barrier integrity and reduces AD-associated inflammation (Figures 4A,B). In the upper row (control or DNCB-induced AD model), strong 4-HNE staining in the epidermal and dermal layers indicates increased lipid peroxidation and oxidative stress. In contrast, the bottom row (like the CVE treatment group) shows reduced 4-HNE staining, suggesting that CVE alleviates oxidative stress and lipid peroxidation in the dorsal skin tissue (Figure 4C).

The process of qualitative analysis involves the identification of distinct plant metabolites within the extract based on their mass-to-charge ratios, elucidated through LC-QTOF-MS-MS analysis of phenolic plant metabolites. LC-QTOF-MS-MS analysis identified several plant metabolites in CVE, including cinnamic acid, trans-cinnamic acid, cinnamaldehyde, and O-methoxy cinnamaldehyde. These plant metabolites have been previously reported to exhibit anti-inflammatory and antioxidant properties, further supporting the observed effects of CVE (Figure 5). These constituents are consistent with previously reported chemical profiles of *CVE*, in which cinnamic acid, trans-cinnamic acid, cinnamaldehyde, and related phenolic derivatives have been described as major metabolites (Pagliari et al., 2023). The plant metabolites detected in our LC-QTOF-MS analysis are therefore consistent with previously reported chemical profiles of CVE. These metabolites contribute to protection against oxidative stress and mitigate inflammation by modulating various cellular pathways and decreasing the synthesis of inflammatory mediators (Pagliari et al., 2023). Many of these plant metabolites possess known anti-inflammatory and antioxidant activities, supporting the biological effects observed in our study. Collectively, these findings suggest that the botanical drug identified in CVE contribute to protection against oxidative stress and attenuation of inflammation through modulation of NF-κB–related pathways.

**FIGURE 6:**
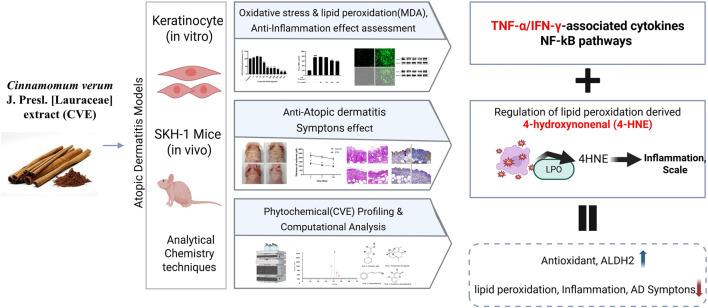
Schematic illustration of the CVE was evaluated using *in vitro* keratinocytes, *in vivo* SKH-1 mice. CVE attenuated atopic dermatitis (AD) by suppressing oxidative stress and NF-κB-mediated inflammatory signaling, reducing inflammatory mediators, and regulating lipid peroxidation-derived 4-hydroxynonenal (4-HNE). These results enhanced cellular antioxidant capacity, increased ALDH2 expression, and decreased lipid peroxidation and inflammation, ultimately ameliorating AD symptoms.

Overall, our results indicate that CVE exerts potent antioxidant and anti-inflammatory effects through multiple mechanisms, including enhancement of aldehyde detoxification, suppression of NF-κB signaling, and inhibition of lipid peroxidation (Figure 6). These findings suggest that CVE may serve as a natural therapeutic agent for the prevention and treatment of inflammatory skin conditions such as AD. Further studies are warranted to explore the clinical applicability of CVE in human subjects and to elucidate its precise molecular mechanisms in greater detail. This study demonstrated the protective effects of CVE against oxidative stress, lipid peroxidation, and inflammation in both cellular and animal models. Our findings provide strong evidence that CVE possesses antioxidant and anti-inflammatory properties, making it a potential candidate for therapeutic intervention in skin-related inflammatory disorders such as AD.

## 5 Conclusion

This study shows that CVE has significant antioxidant and anti-inflammatory properties, successfully improving symptoms resembling AD in cellular and animal models. The application of CVE resulted in a notable reduction in oxidative stress and lipid peroxidation, as demonstrated by lower levels of MDA and 4-HNE, alongside an increase in the expression of ALDH2, which contributed to the enhancement of aldehyde detoxification. Moreover, CVE suppressed NF-κB signaling by inhibiting IκBα and p65 phosphorylation, subsequently decreasing the production of pro-inflammatory molecules including COX-2 and iNOS. Analysis via LC-QTOF-MS/MS revealed the presence of various plant metabolites in CVE that possess known antioxidant and anti-inflammatory properties. In conclusion, these findings indicate that CVE botanical drug could potentially serve as an effective natural therapeutic treatment for inflammatory skin conditions like AD. Future studies will focus on the isolation and characterization of specific metabolites and on validating their biological activities using targeted experimental approaches.

## Data Availability

The original contributions presented in the study are included in the article/Supplementary Material, further inquiries can be directed to the corresponding authors.
