# Supplementary material for: Cinnamomum verum extract as a potential therapeutic for atopic dermatitis through reducing lipid peroxidation and oxidative stress
Source: Front Pharmacol. 2026 Jan 12;16:1714816. doi: 10.3389/fphar.2025.1714816 (PMC12833377; doi:10.3389/fphar.2025.1714816)
Supplement: Supplementary file 1 [file Supplementaryfile1.docx]

**Supplementary Materials**


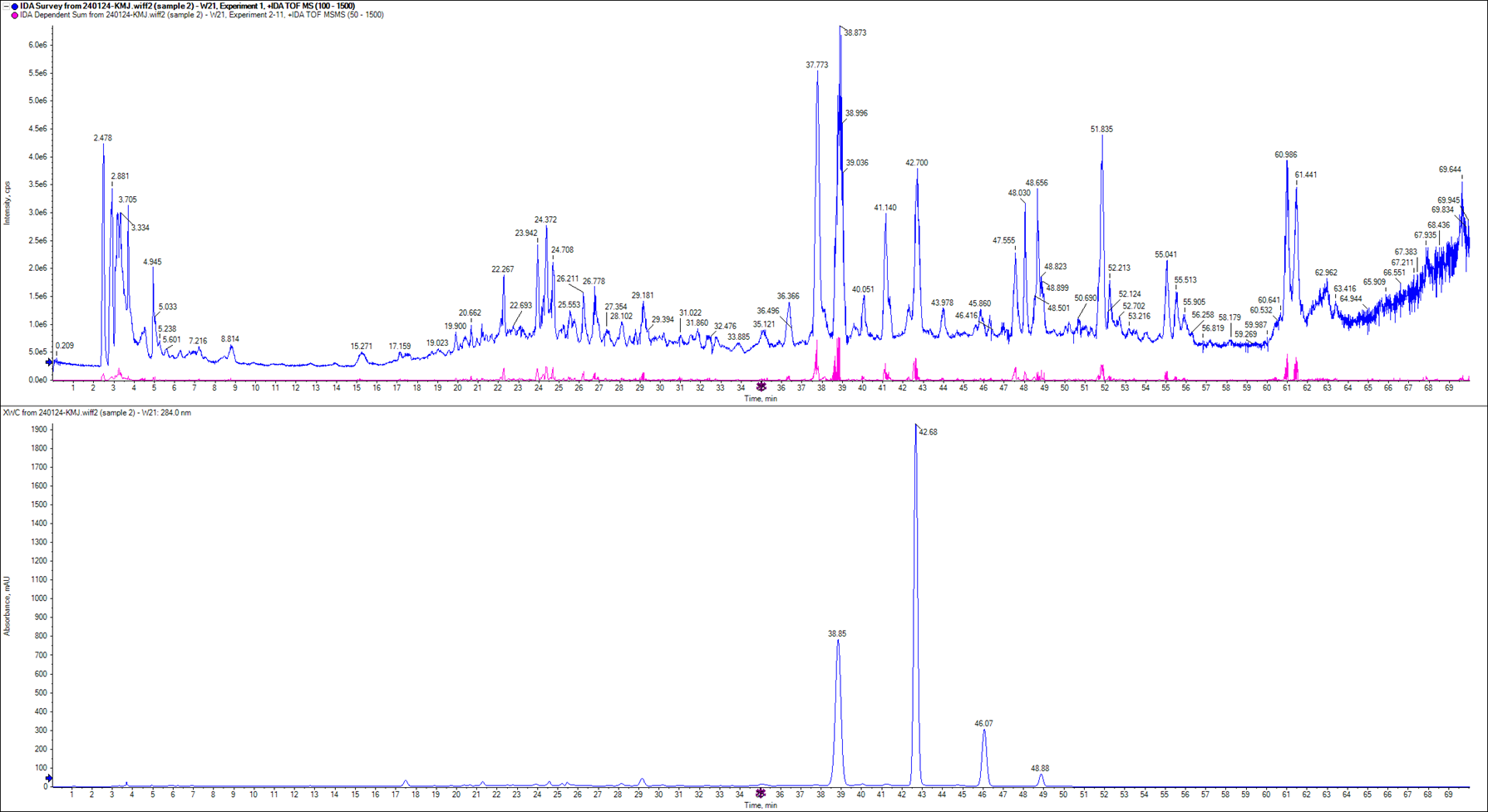


**Supplementary Figure 1. Precursor positive ion full scan spectra of CVE with LC-MS-MS.**


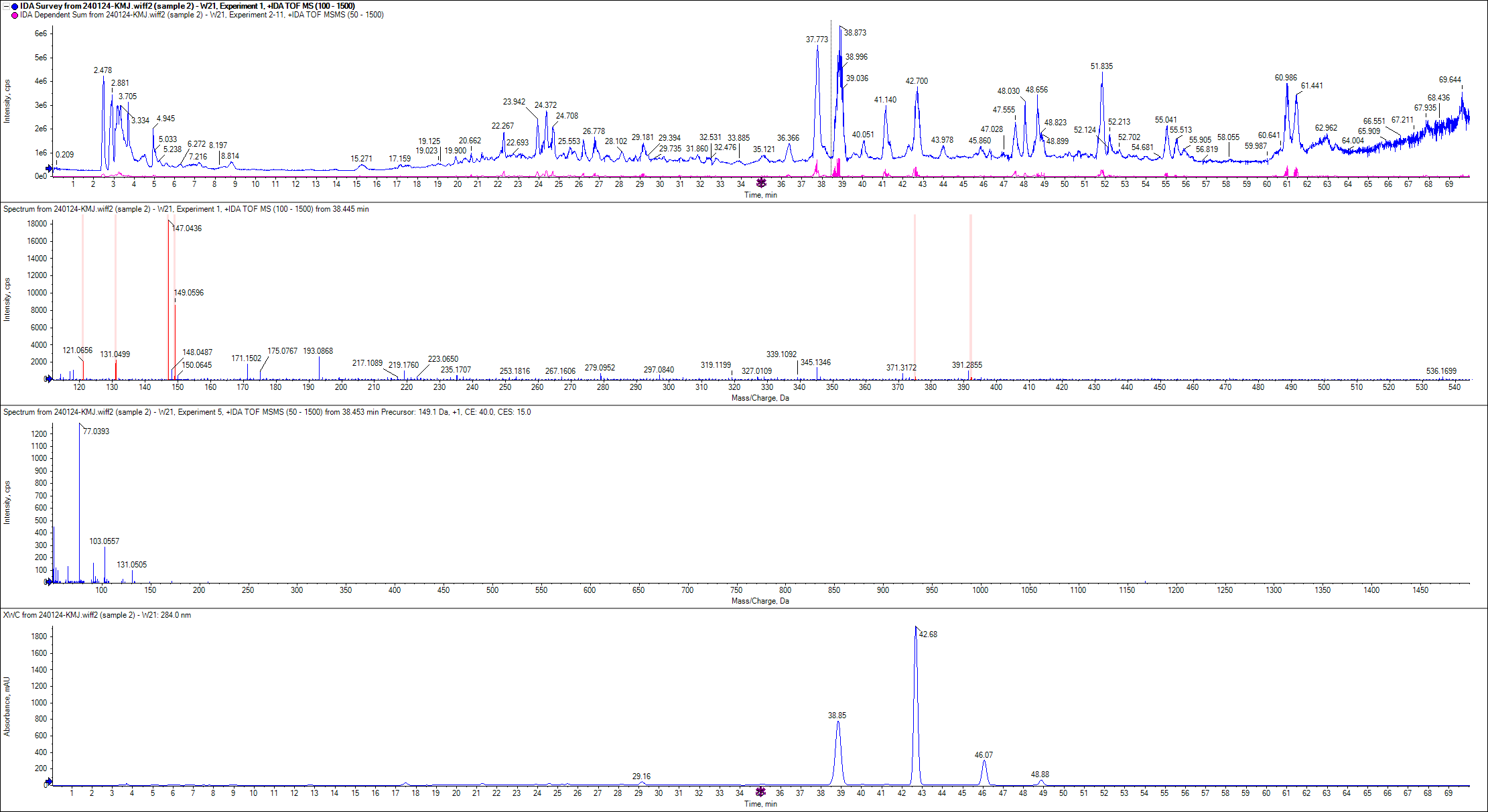


**Supplementary Figure 2. LC–MS/MS Spectra and Peak 1 Cinnamic acid.**


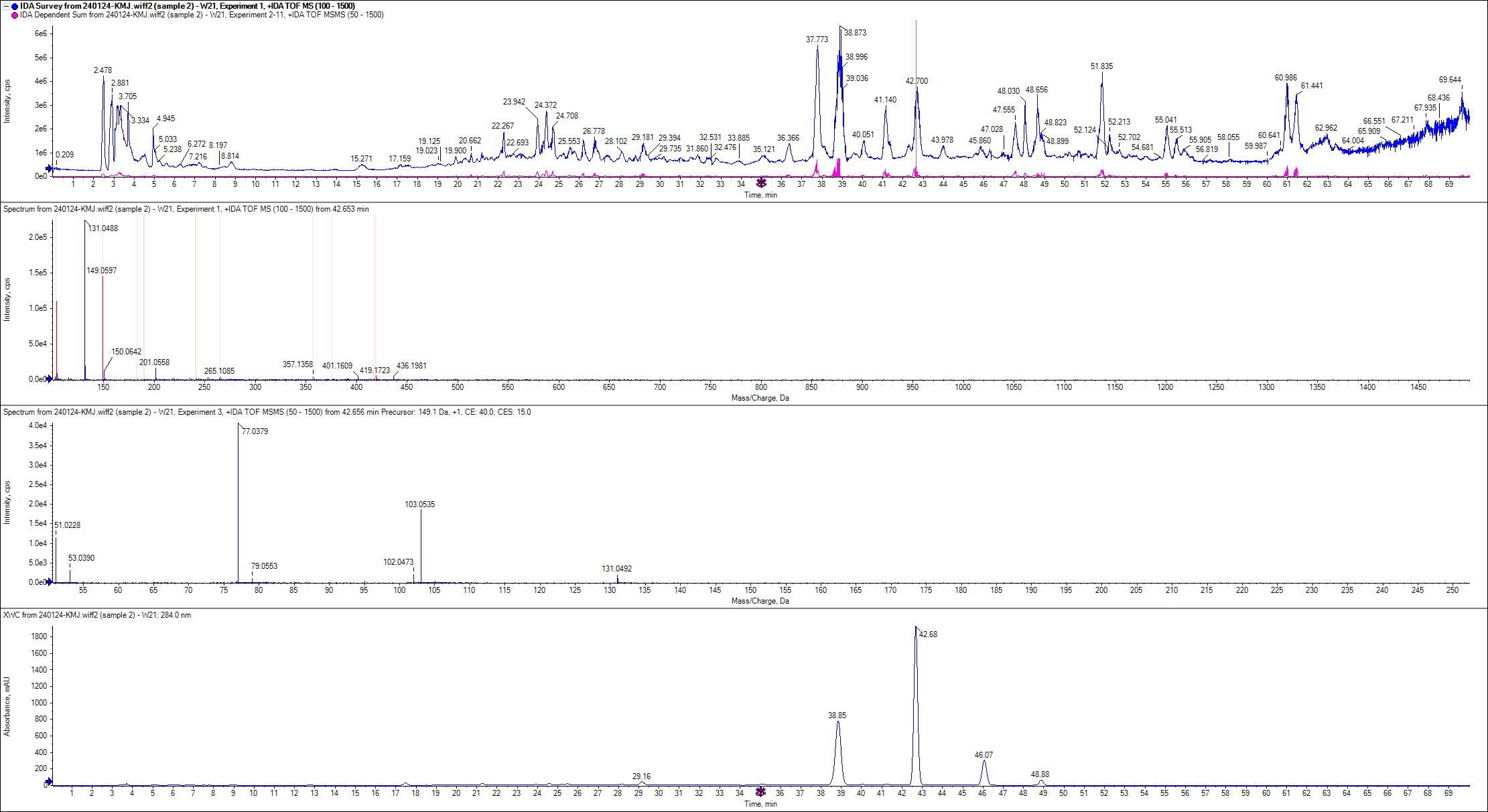


**Supplementary Figure 3. LC–MS/MS Spectra and Peak 2** **Trans-cinnamic acid.**

**
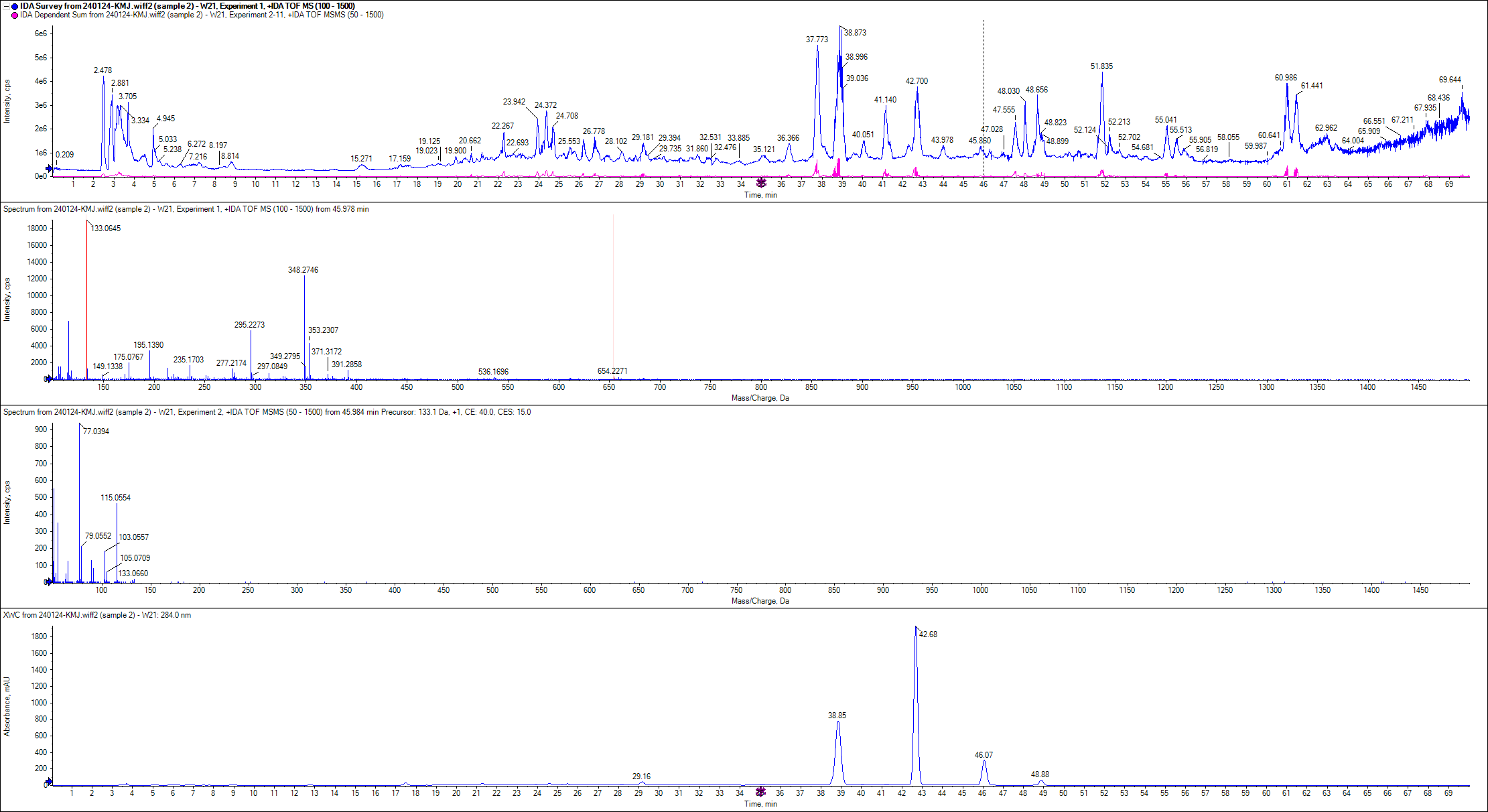
**

**Supplementary Figure 4. LC–MS/MS Spectra and Peak 3 Cinnamaldehyde**.

**
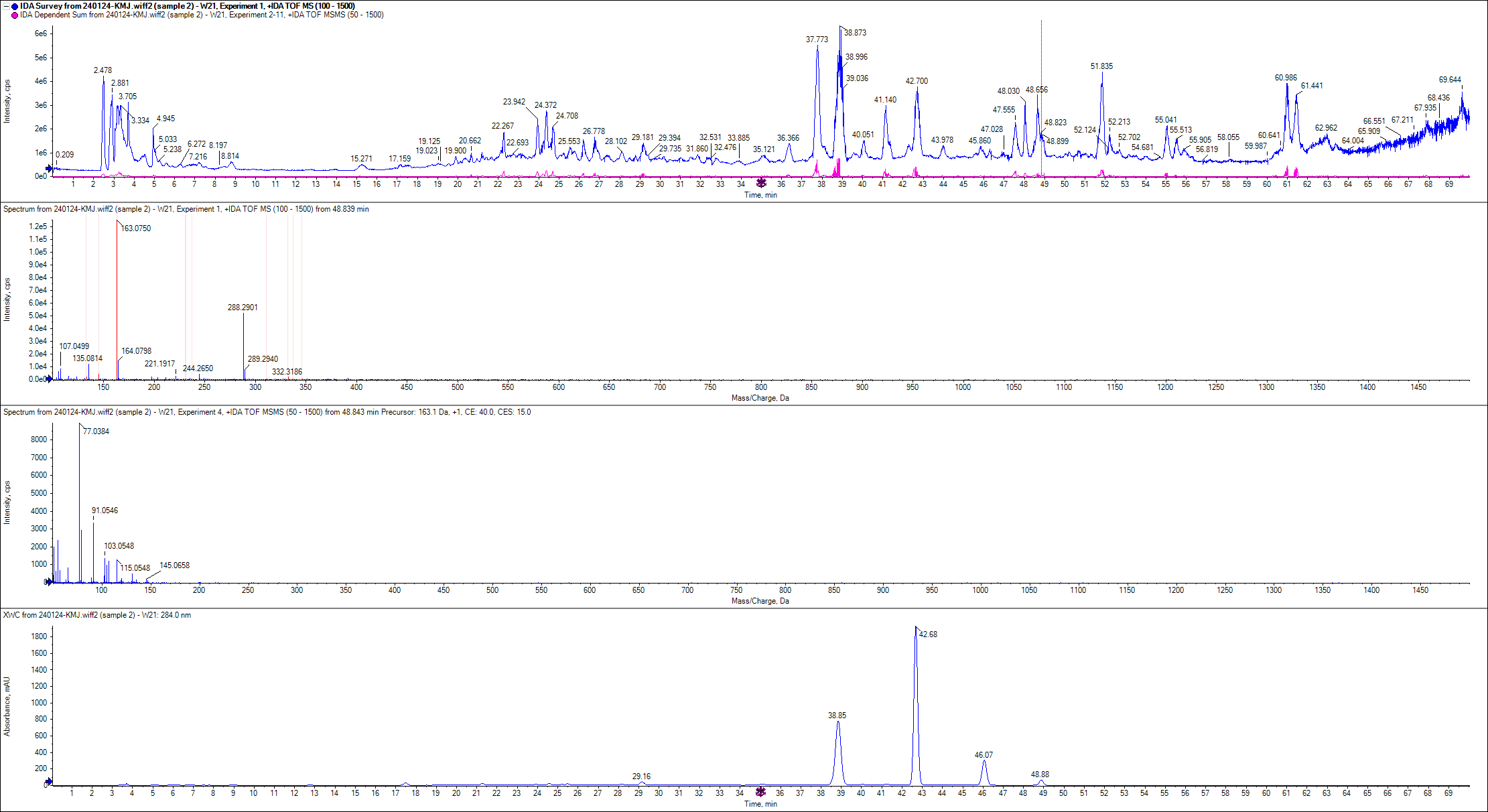
**

**Supplementary Figure 5. LC–MS/MS Spectra and Peak 4. O-methoxy cinnamaldehyde**.
